# Clay hypoplasticity coupled with small-strain approaches for complex cyclic loading

**DOI:** 10.1007/s11440-023-02087-w

**Published:** 2023-11-16

**Authors:** Gertraud Medicus, Merita Tafili, Manuel Bode, Wolfgang Fellin, Torsten Wichtmann

**Affiliations:** 1 Universität Innsbruck, Austria, Computational and Experimental Soil Mechanics, Unit of Geotechnical Engineering, Technikerstr. 13, 6020 Innsbruck, Austria; 2 Ruhr-University Bochum, Germany, Chair of Soil Mechanics, Foundation Engineering and Environmental Geotechnics, Universitätsstraße 150, 44801 Bochum, Germany

**Keywords:** Clay, Cyclic loading, Hypoplasticity, Intergranular strain, ISA, Small-strain stiffness, 0000, 1111

## Abstract

Constitutive models that are able to accurately predict cyclic soil behaviour are crucial for finite element design of offshore foundation or railway embankments. Basic hypoplastic models introduce the history of loading in state variables such as the stress and void ratio and are therefore incapable of describing small-strain stiffness and cyclic loading. In this work, clay hypoplasticity is extended with a modified intergranular strain proposed by Duque et al. [3]. The new model is compared to the one coupled previously with ISA based on unconventional as well as complex cyclic loading paths. Abilities and limitations of the models are addressed: (i) showing that both models predict a reduction in strain accumulation with an increasing number of cycles. (ii) For both models pronounced over- and undershooting effects can occur for certain cyclic loading paths and certain parameters. Despite the consensus in the literature, the results show that a yield surface in the (intergranular) strain space is not sufficient to ban these effects. Furthermore, the models’ predictive capabilities are verified with simulations of monotonic and cyclic tests of Lower Rhine clay.

## Introduction

Hypoplasticity is a constitutive framework introduced by Kolymbas [18] as a counterpart to elastoplasticity for describing the soil behaviour using only one tensorial equation. Since the introduction of this framework many improvements and verifications have been proposed for sand [13, 15, 30, 40, 41] as well as for clays [12, 14, 22, 26, 36] testifying good accordance to experimental observations with monotonic loading paths. A fundamental drawback of simple hypoplastic models lies on the significant underestimation of the small-strain stiffness and thus the basic model cannot be used for cyclic loading. In addition, the recent discussion by Kolymbas [19] and Duque et al. [6] demonstrates the interest in constitutive modelling of cyclic loading.

To improve the performance of hypoplastic models in the range of small strains as well as under cyclic loading, a state variable called intergranular strain (IGS) was introduced by Niemunis and Herle [27]; Niemunis [25]. This tensorial variable should represent the deformation of an interface layer between soil particles and thus provides an increased stiffness at loading reversals and small load cycles. The IGS approach indicated good performance in simulating the stiffness increase upon reversal loading but failed to reproduce the memory effects upon reloading paths. In an attempt to overcome this shortcoming, Fuentes and Triantafyllidis [10] reformulated the IGS to an elastoplastic framework inside the intergranular strain space and named it intergranular strain anisotropy (ISA) model for sand. It was claimed that several salient features were obtained, among them the existence of an elastic locus related with a strain amplitude, which should enable the simulation of memory effects and the simulation of the stiffness increase upon reversal loading. ISA was further extended to capture the influence of a larger number of consecutive cycles before reaching the critical state and thus reducing the strain or pore water pressure accumulation by Poblete et al. [28]. This version of ISA has been incorporated in different works for sand comprising benchmark simulations [11, 17, 20, 39], for clays without rate dependency [7, 8] as well as incorporating the rate dependency of fine-grained soils [9, 32, 35]. On the other hand, IGS was improved by Wegener and Herle [38] to describe accumulation effects more realistically, and subsequently, a similar modification as proposed by Poblete et al. [28] for ISA was incorporated into the IGS by Duque et al. [3] to improve the simulations under larger number of repetitive cycles. The advantages of IGS as well as ISA were used for the improvement of non-hypoplastic models by Bode et al. [1] and Tafili et al. [34].

Nevertheless, many limitations of advanced constitutive models for soils have been detected in novel works regarding cyclic loading paths. Duque et al. [4, 5] investigated eight advanced constitutive models (four for sand and four for clay) suitable for cyclic loading and addressed the different models’ limitations. Among other limitations, the problem of stress overshooting is highly topical in numerical soil mechanics for both hypoplasticity (Duque et al. [5]) as well as elastoplasticity (Dafalias and Taiebat [2]). Hypoplastic models using small-strain extensions, consider boundaries in (intergranular) strain space for small-strain loading, but no boundaries in stress space. As a result, the issue of stress overshooting could alleviate as shown in Duque et al. [5] as well. Nevertheless, the simulation results of the HP+ISA model in Duque et al. [5] do not show overshooting, which contradicts the findings of this paper. In addition, Duque et al. [5] investigated strain accumulation for applied cyclic loading stress loops. Thereby, HP+ISA could well predict a decrease in volumetric strain accumulation with increasing number of cycles, while HP+IGS (IGS according to Niemunis and Herle [27]) failed. However, these investigations where restricted to sands. In Duque et al. [4] four constitutive models for clays have been examined, namely the anisotropic hypoplastic model by Fuentes et al. [8], the SANICLAY-B elastoplastic model by Seidalinov and Taiebat [29], the constitutive Anamnesis model by Tafili et al. [33] and the three surface kinematic hardening model proposed by Stallebrass and Taylor [31] with transverse isotropic elasticity. This study was mainly focused to advanced constitutive models with stiffness anisotropy and cyclic loading and does not address the stress overshooting problem with clay models. The most applied hypoplastic model for fine-grained soils is the model according to Mašín [22], which has however not been investigated by Duque et al. [4]. Furthermore, it was never extended with the IGS according to Duque et al. [5] to reproduce a larger number of cycles. It is therefore of great interest to analyse and address possible shortcomings of this model.

For application purposes simple models with reasonable implementation and calibration effort are required. Simultaneously, high quality prediction under monotonic as well as cyclic loads are expected.

The present paper extends the simple and widely used clay hypoplasticity model according to Mašín [22] with the last developments of the two intergranular strain concepts: ISA according to Poblete et al. [28] and IGS according to Duque et al. [3]. A detailed qualitative and quantitative analysis is presented, whereby advantages and disadvantages of the models considering the behaviour of clays are addressed. The stress overshooting phenomenon can be problematic in practical applications of constitutive models, due to many recurring small stress and strain cycles in the lifetime of diverse foundations. It is shown that the results of Duque et al. [5] for applied cyclic stress loops for hypoplasticity with IGS do not hold when the modification to IGS presented by Duque et al. [3] is employed. In particular, the results show that overshooting may pose a serious issue not only for IGS (for which it is a known shortcoming), but also for ISA if the parameters are chosen unfavourably. Finally, both models are calibrated on laboratory data and their predictions are compared with experiments of Lower Rhine Clay [37]. The qualitative simulation results can furthermore serve as idea for future experimental and numerical research activities. The notation is used as in Tafili et al. [34] and Bode et al. [1] and included for completeness in Appendix A.

## Constitutive models

### Clay hypoplasticity (HP)

Clay hypoplasticity by Mašín [22] is a constitutive model that includes concepts from critical state soil mechanics and an explicit formulation of the so-called asymptotic state boundary surface. The cross section of the critical stress surface coincides to Matsuoka and Nakai [21]; Nakai et al. [24]. The objective stress rate $\mathring{\varvec{T}}$ follows the general formulation of hypoplastic models:

1 $$\begin{aligned} \mathring{\varvec{T}} &= f_s\left( \pmb {\mathcal {L}}:\varvec{D}+f_d\varvec{N}\Vert \varvec{D}\Vert \right) \end{aligned}$$

with the stretching tensor $\varvec{D}$, barotropy factor (influence of mean stress) $f_s$, pyknotropy factor (void ratio influence) $f_d$ and the respective fourth- and second-order stress and stretching-dependent tensors $\pmb {\mathcal {L}}$ and $\varvec{N}$. Considering these dependencies, the model can well predict different monotonic loading paths, but has limitations for predictions of small-strain stiffness as well as cyclic loading paths. To overcome these shortcomings, mainly two extensions of hypoplastic models have been proposed in the literature.

### Clay hypoplasticity enhanced with ISA (HP+ISA)

**Fig. 1:**
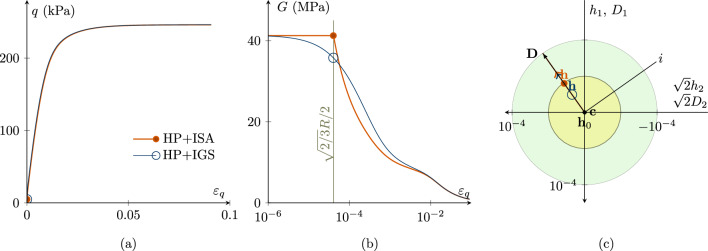
Cu test with $\varvec{h}_{0}=\varvec{c}_0=\textbf{0}$; $\chi _0=\chi _\text {max}=1$; $p'_0=200$ kPa; $\text{ OCR}_0=2.5$. Simulations with HP+ISA and HP+IGS. (a) deviatoric stress *q*—deviatoric strain $\varepsilon _q$ plot, (b) the secant shear stiffness *G*—deviatoric strain $\varepsilon _q$ plot, (c) Rendulic planes of intergranular strain $\varvec{h}$ and stretching $\varvec{D}$ for the state indicated by circles in (a) and (b). HP+ISA behaves purely elastic until $\varepsilon _q=\sqrt{2/3}\cdot R/2$

The ISA plasticity was initially developed for sand by Fuentes and Triantafyllidis [10]. However, the most well-known and established version is the one developed by Poblete et al. [28], which will be used in this work. Both intergranular strain extensions incorporate at least one new tensorial state variable, the intergranular strain tensor $\varvec{h}$, into the model. The main difference of ISA is the ISA yield surface with the radius *R*/2 formulated in the intergranular strain space as will be shown through the qualitative simulations in the next section, for instance see Fig. 1. Besides the yield surface, the model includes a bounding intergranular strain surface considering another state variable in the intergranular strain space called the kinematic hardening tensor $\varvec{c}$. The hypoplastic models response coupled with ISA is rendered (hypo)elastic (depending on the elastic tangent stiffness introduced in the constitutive relations) after a loading reversal as long as $\Vert \varvec{h}\Vert \le R$, hence if $\varvec{h}$ is inside the yield surface. Within reloading, when $0<\Vert \varvec{h}\Vert < R$, the models response is in transition between (hypo)elasticity and hypoplasticity. The latter is reached at fully mobilized states, hence at the bounding intergranular strain $\Vert \varvec{h}\Vert =R$ and $\Vert \varvec{c}\Vert =R/2$ and if stretching $\varvec{D}$ and $\varvec{h}$ point in the same direction.

To reduce the strain accumulation under drained conditions as well as the pore water pressure accumulation under undrained conditions for increasing number of consecutive cycles before reaching the critical state, Poblete et al. [28] introduced a scalar state variable responsible for the cyclic history obeying the following evolution equation:

2 $$\begin{aligned} \dot{\varepsilon }_a=C_a/R\left( 1-y_h-\varepsilon _a\right) \Vert \varvec{D}\Vert . \end{aligned}$$

The intergranular strain scalar function $y_h$ quantifies the distance between $\varvec{h}$ and the bounding surface indicating monotonic loading for $y_h=1$ and a vanishing $\varepsilon _a$. Inside the yield surface, hence under unloading or small reloading as well as for a large number of consecutive cycles far from the critical state $y_h\rightarrow 0\Rightarrow \varepsilon _a\rightarrow 1$. The new state variable is then used to modify the scalar function controlling the plastic accumulation rate of the model:

3 $$\begin{aligned} {\text{ extension } \text{(I) }}\quad \chi =\chi _0+\varepsilon _a\,(\chi _\text {max}-\chi _0) \end{aligned}$$

with the material parameters $\chi _0$ and $\chi _{max}$. The two limit cases, $\chi =\chi _0$ indicates monotonic loading, while $\chi =\chi _\text {max}$ reduces the cumulative rates depending on the loading condition significantly.

### Clay hypoplasticity enhanced with IGS according to Duque et al. [3] (HP+IGS)

Duque et al. [3] extended the intergranular strain concept according to Niemunis and Herle [27] with the above described $\varepsilon _{a}$-formulation from Poblete et al. [28] that allows a transition from $\chi _0$ to $\chi _\text {max}$ (extension (I)) and applied it to sand hypoplasticity by von Wolffersdorff [40]. In this work, clay hypoplasticity [22] is extended with the modified IGS according to Duque et al. [3]. In addition, with IGS according to Duque et al. [3], it is possible to interpolate the linear and the nonlinear term in different ways, by using two different exponents, comparable to what was proposed by Wegener and Herle [38]. This is done using $\gamma$ (extension (I)) in addition to $\chi$ as exponents in the material stiffness matrix^1^:

4 $$\begin{aligned} \begin{aligned} \pmb {\mathcal {M}} & = [y_h^\chi m_T+(1-y_h^\chi )m_R]\pmb {\mathcal {L}} \\&+{\left\{ \begin{array}{ll} y_h^\chi (1-m_T)\left( \pmb {\mathcal {L}}:\varvec{h}^0\right) \otimes \varvec{h}^0+y_h^\gamma \varvec{N}\otimes \varvec{h}^0~~~~\text {for}~\varvec{h}^0:\varvec{D}>0,\\ y_h^\chi (m_R-m_T)\left( \pmb {\mathcal {L}}:\varvec{h}^0\right) \otimes \varvec{h}^0~~~~\text {for}~ \varvec{h}^0:\varvec{D}\le 0 \end{array}\right. } \end{aligned} \end{aligned}$$

with the normalized length of the intergranular strain tensor^2^$y_h=||\varvec{h}||/R$ and with $\gamma _\chi$ as an additional parameter in $\gamma =\chi \cdot \gamma _\chi$. As for ISA, the variation of $\chi$ in Eq. 3 is carried out using the additional state variable $\varepsilon _{a}$. Similar to ISA, the variable $\varepsilon _{a}$ follows an evolution equation with

5 $$\begin{aligned} {\text{ extension } \text{(II) }}\quad \dot{\varepsilon }_a=C_a/R\left( 1-y_h^\gamma -\varepsilon _a\right) \Vert \varvec{D}\Vert . \end{aligned}$$

Whenever the current state is within the range of intergranular strain, $y_h$ becomes less than 1, leading to an increase in $\dot{\varepsilon }_\text {a}$. For monotonic deformation, as soon as the intergranular strain is mobilized, $y_h = 1$ and using the accumulated value of $\varepsilon _\text {a}$, $\dot{\varepsilon }_\text {a}$ becomes negative and $\varepsilon _\text {a}$ decreases again. Thus, a monotonic deformation *forgets* that there was once an accumulation of load cycles. Extension (I) can be deactivated if $\chi _\text {max}$ is set equal to $\chi _0$. Extension (II) can be deactivated if $\gamma _\chi$ is set to 1. For example for a cyclic, undrained triaxial test, the following applies: the larger $\gamma _\chi$ is the lower is the accumulation rate of either the pore water pressure or the axial strain. The influence of $\gamma _\chi$ is further investigated quantitatively in Sect. 4.3. Without the extensions (I) and (II), the original intergranular strain concept according to Niemunis and Herle [27] is obtained. A more detailed description can be found in Niemunis and Herle [27]; Duque et al. [3]; Wegener and Herle [38].

## Qualitative analysis

In this section, similarities and differences between the two models are addressed by qualitative simulations. The initial conditions of the simulations are summarized in Table 2. The initial density can be characterized through a starting value for the initial void ratio $e_0$ or the initial OCR_0_. From OCR_0_, $e_0$ is obtained as follows:

6 $$\begin{aligned} e_0=\exp \left( N-\lambda ^*\ln (\text {OCR}_0\cdot p_0')\right) -1 \end{aligned}$$

### Calibration

The parameters for clay hypoplasticity and the small-strain extensions ISA and IGS are listed in Table 1. The material constants were selected in a way that makes it feasible to demonstrate both the conceptual similarities and contrasts that the models may evidence. These differences and similarities are also valid for other sets of parameters, even though they might be less pronounced.

**Table 1:** Default parameter set for clay hypoplasticity (HP) Mašín [22], coupled with IGS or ISA

Model	$\varphi _c$	*N*	$\lambda ^*$	$\kappa ^*$	$\nu$	$m_T=m_R$	*R*	$\beta _R$	$\chi _0$	$\chi _\text {max}$	$C_a$	$\gamma _\chi$
HP+ISA	$25^\circ$	1	0.1	0.01	0.2	5	$1\times 10^{-4}$	0.5	1	20	0.018	−
HP+IGS										9	0.007	1

### Triaxial element tests

In this section, monotonic and cyclic undrained triaxial (cu) tests are simulated to illustrate the models’ qualitative predictions.

**Table 2:** Initial conditions, qualitative analysis

Test	$\varvec{T}_0$ (kPa)	OCR_0_	$\varvec{h}_0$	$\varvec{c}_0$
Cu test, Fig. 1	$-200\cdot \varvec{I}$	2.5	${0}$	${0}$
Cu test, Fig. 2	$-200\cdot \varvec{I}$	2.5	${0}$	${0}$
Cu test, Fig. 3	$-200\cdot \varvec{I}$	2.5	$$\sqrt{2/3} R\begin{pmatrix} 1 &{} 0 &{} 0 \\ 0 &{} -1/2 &{} 0 \\ 0 &{} 0 &{} -1/2 \\ \end{pmatrix}$$	$\varvec{h}_0/2$
Stress loop cycles, Fig. 4	$$\begin{pmatrix} -260 &{} 0 &{} 0 \\ 0 &{} -150&{} 0 \\ 0 &{} 0 &{} -150 \\ \end{pmatrix}$$	3	$$R\begin{pmatrix} -1 &{} 0 &{} 0 \\ 0 &{} 0 &{} 0 \\ 0 &{} 0 &{} 0 \\ \end{pmatrix}$$	$\varvec{h}_0/2$
$p'=\,$const cycles, Fig. 5	$$\begin{pmatrix} -260 &{} 0 &{} 0 \\ 0 &{} -150&{} 0 \\ 0 &{} 0 &{} -150 \\ \end{pmatrix}$$	3	$$R\begin{pmatrix} -1 &{} 0 &{} 0 \\ 0 &{} 0 &{} 0 \\ 0 &{} 0 &{} 0 \\ \end{pmatrix}$$	$\varvec{h}_0/2$
$p'=\,$const cycles, Fig. 6	$$\begin{pmatrix} -260 &{} 0 &{} 0 \\ 0 &{} -150&{} 0 \\ 0 &{} 0 &{} -150 \\ \end{pmatrix}$$	3	${0}$	${0}$
Stress loop cycles, Fig. 7	$$\begin{pmatrix} -240 &{} 0 &{} 0 \\ 0 &{} -187&{} 0 \\ 0 &{} 0 &{} -133 \\ \end{pmatrix}$$	3	$$R\begin{pmatrix} -0.91 &{} 0 &{} 0 \\ 0 &{} -0.34 &{} 0 \\ 0 &{} 0 &{} 0.24 \\ \end{pmatrix}$$	$\varvec{h}_0/2$
Cu test, Figs. 8, 9, 10	$-200\cdot \varvec{I}$	3	$-R\varvec{I}/\sqrt{3}$	$\varvec{h}_0/2$

#### Monotonic cu tests

Figure 1 shows a monotonic undrained triaxial test without initializing the intergranular strain, i.e. $\varvec{h}_0=\varvec{0}$. Thereby, Fig. 1a presents the deviatoric stress versus deviatoric strain curve, whereas in Fig. 1b, the secant shear stiffness degradation is depicted. The initial small-strain response for HP+ISA is purely elastic up to $\varepsilon _q=\sqrt{2/3}\cdot R/2$ followed by a transition between ISA and hypoplasticity until the purely hypoplastic models’ response is reached when the intergranular strain lies at the bounding surface (green surface in Fig. 1c). In Fig. 1c, the Rendulic plane of stretching and intergranular strain space is shown. The location of the purely elastic ISA yield surface (yellow circle) corresponds to the current state marked with $\bullet$. Different to that the response of HP+IGS is not purely elastic, not even for very small strains. This fact indicates that at least for the herein chosen parameters, HP+IGS might result in stress accumulation under very small-strain cyclic loading, whereas HP+ISA is not expected to. This difference only occurs in the small-strain range ($\varepsilon _q=10^{-6}$ to $10^{-4}$) as displayed in Fig. 1c. However, for a deviatoric strain larger $10^{-4}$, the models’ response is similar, hence it is anticipated that under monotonic loading, and they will exhibit the same behaviour.

**Fig. 2:**
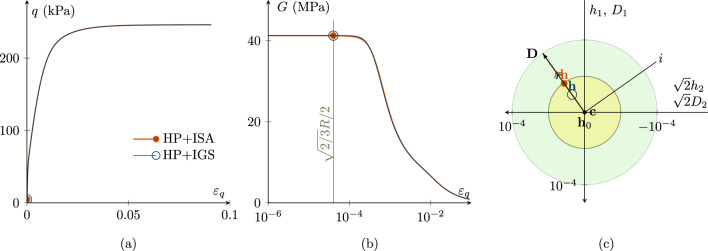
Cu test with $\varvec{h}_{0}=\varvec{c}_0=\textbf{0}$; $\chi _0=\chi _\text {max}=20$ for ISA and $\chi _0=\chi _\text {max}=9$ for IGS; $p'_0=200$ kPa; $\text{ OCR}_0=2.5$. Simulations with HP+ISA and HP+IGS then practically coincide. **a** deviatoric stress *q*—deviatoric strain $\varepsilon _q$ plot, **b** the secant shear stiffness *G*—deviatoric strain $\varepsilon _q$ plot, **c** Rendulic planes of intergranular strain $\varvec{h}$ and stretching $\varvec{D}$ for the state indicated by circles in **a** and **b**

In addition, Fig. 2 shows the same simulations with $\chi _0=\chi _\text {max}=20$ for ISA and 9 for IGS (compare in Fig. 1, $\chi _0=\chi _\text {max}=1$ holds for both models). All other parameters and initial conditions coincide with the ones chosen in the simulation in Fig. 1. Both models’ predictions almost coincide, due to the increase in $\chi$. HP+ISA again behaves purely elastic until $\varepsilon _q=\sqrt{2/3}\cdot R/2$. Nevertheless, first reaction of HP+IGS is not fully elastic and is a result of interpolating between (hypo)elasticity and hypoplasticity. Still, because $\chi _0=\chi _\text {max}$ is set to a large value, the initial shear modulus is maintained at a level comparable to that of ISA. Similar observations as in Figs. 1 and 2 have been made for the model barodesy coupled with intergranular strain concepts in Tafili et al. [34].

#### Cyclic cu test

**Fig. 3:**
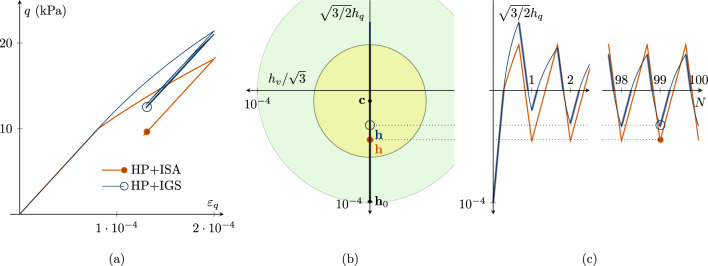
Cyclic cu test with 100 small-strain cycles: The models’ predictions are similar. Due to the transition from $\chi _0$ to $\chi _\text {max}$, stress accumulation is also low for HP+IGS. **a** Deviatoric strain $\varepsilon _q$—deviatoric stress *q* plot, **b** $h_v/\sqrt{3}$-$\sqrt{3/2}h_q$ plane of intergranular strain space with the location of the elastic ISA yield surface, that corresponds to the current state $\bullet$, **c** deviatoric intergranular strain $\sqrt{3/2}h_q$ development with ongoing cycles *N*. For HP+ISA, the response is purely elastic. For HP+IGS, the bold blue line indicates the elastic response and the thin line indicates interpolation between elasticity and hypoplasticity (colour figure online)

Figure 3 contains cyclic undrained triaxial simulations. The initial stress is isotropic at $p=200$ kPa and the intergranular strain is initialized as if the sample just went through an isochoric triaxial unloading, and is now subjected to isochoric triaxial loading, see Table 2. These conditions might occur in the reality after for an earthquake loading. Afterwards, the numerical sample is initially sheared up to $\varepsilon _q=2\cdot R$ followed by 100 deviatoric strain cycles with an amplitude of $\Delta \varepsilon _q=7\cdot 10^{-5}$. In Fig. 3, only the first and last cycles are depicted, whereby in (a) the deviatoric strain $\varepsilon _q$—deviatoric stress *q* is visualized and in (b) the $h_v/3$-$\sqrt{3/2}h_q$ plane of intergranular strain space is presented. The location of the ISA yield surface corresponds to the current state marked with $\bullet$ in all plots. Figure 3c shows the deviatoric intergranular strain $\sqrt{3/2}h_q$ development with the number of cycles *N*. For HP+ISA, the cyclic response is purely elastic, as the strain amplitude is within the purely elastic yield surface, see Fig. 3b. For HP+IGS, the response is purely elastic after each loading reversal, but just as long as $\varvec{h}:\varvec{D}<0$.^3^ In Fig. 3c, the bold blue line indicates the elastic response ($\varvec{h}:\varvec{D}<0$) and the thin line indicates interpolation between elasticity and hypoplasticity ($\varvec{h}:\varvec{D}>0$). At the very beginning, the transition causes some accumulation for the deviatoric stress *q*. This accumulation is, however, relatively small, as the increase from $\chi _0$ to $\chi _\text {max}$ reduces the accumulation also for HP+IGS. Therefore, it can be concluded that for higher values of $\chi$, the models’ response can be very similar even though HP+IGS does not include a purely elastic yield surface.

### Complex stress and strain paths

Constitutive models are often investigated for conventional and axisymmetric tests only. This section is therefore focused on unconventional cyclic loading paths in order to estimate the models’ abilities and limitations.

#### Axisymmetric multidirectional stress loop

Poblete et al. [28] carried out experiments in which cyclic stress loops were applied to sand specimens. The strain accumulation decreased with increasing number of cycles when the stress state was far away from the critical state, similar to what was shown for hypoplasticity coupled with ISA [5, 28]. Duque et al. [5] showed that for hypoplasticity coupled with the intergranular strain concept according to Niemunis and Herle [27], strain accumulation is constant for ongoing cyclic loading and thus highly overestimates the strain accumulation.

**Fig. 4:**
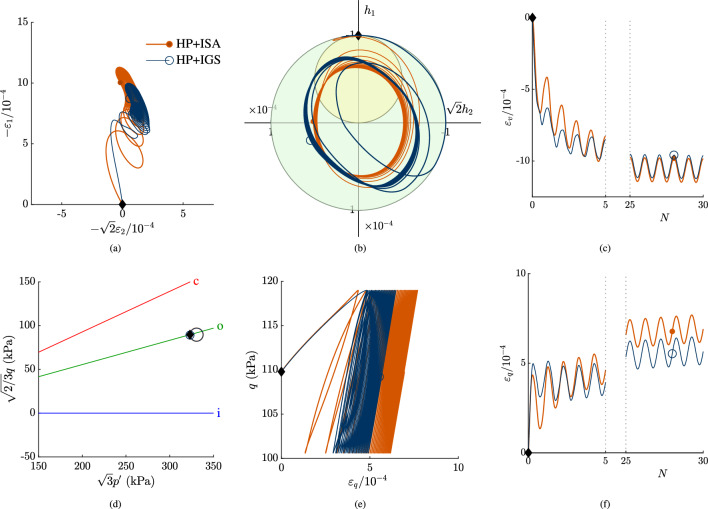
Stress circle in clockwise direction with an initial OCR$\,=3$, starting at oedometric compression state. The initial state is marked with $\blacklozenge$ in all plots, in **a** the Rendulic plane of strain space is shown, in **b** the Rendulic plane of intergranular strain space is shown, where the ISA yield surface corresponds to the initial state. In **c** the volumetric response with ongoing number of cycles is shown. **d** shows the stress path, where **c** indicates the CSL, o the $K_0$-path and i is the hydrostatic axis. **e** is the *q*-$\varepsilon _q$ plot, In **f** the deviatoric strain with ongoing number of cycles *N* is shown

In Fig. 4, simulations of stress loops with 30 cycles are shown. The applied stress loops are circles in the Rendulic plane, see also Fig. 4d, that are applied in clockwise direction:

7 $$\begin{aligned} \Delta \sigma _1'&= r \sin {t} \end{aligned}$$

8 $$\begin{aligned} \sqrt{2}\Delta \sigma _2'&= r \cos {t}= \sqrt{2}\Delta \sigma _3' \end{aligned}$$

whereby $r=7.5$ kPa in the simulation in Fig. 4 and $0\le t\le 2\pi$. With the initial stress state according to Table 2, the stress loop is obtained: $\sigma _i' = \sigma _{i,\text {0}}'+\Delta \sigma _i'$. The initial stress state corresponds to an axisymmetric, oedometric compression state with initial conditions according to Table 2, see also Fig. 4d. The intergranular strain obeyed the strain direction in a fully mobilized oedometric compression, hence fully mobilized in vertical direction, while the lateral components are $h_2=h_3=0$. The $p'$-*q* plane has to be scaled, in order to show the circular shape of the stress loop, i.e. $\sqrt{3}p'$—$\sqrt{2/3}q$. The applied stress loops result in strain and intergranular strain paths, which follows from an interpolation between elasticity and hypoplasticity, see Fig. 4a and b. The volumetric and deviatoric strain accumulation rate reduces with an increasing number of cycles for both models, see Fig. 4c, e and f. Duque et al. [5] and Poblete et al. [28] state that in order to predict these kind of multidimensional paths, i.e. a reduction in strain accumulation rate with increasing number of cycles, the existence of an elastic locus is necessary. Hereby it is shown, that this feature can be reached with HP+IGS without a purely elastic locus as well. However, the transition from $\chi _0$ to $\chi _\text {max}$ as proposed by Duque et al. [3] and used in HP+IGS is necessary. Comparison between the predictions in Fig. 4 and the results of hypoplasticity for sand coupled with the intergranular strain concept according to Niemunis and Herle [27] presented by Duque et al. [5] illustrates the improvement.

A lower and fixed value for $\chi _0=\chi _\text {max}$ would imply an ongoing strain accumulation and thus overestimation of strain for both models, thus independently of the yield surface of ISA. Therefore, extension (I) is necessary to be included in the HP+IGS in order to reduce accumulation effects with increasing number of cycles. Similar to that larger stress loops that would lead to fully mobilized intergranular strain, would also lead to an ongoing strain accumulation for both models.

#### Uniaxial versus multiaxial loading paths

**Fig. 5:**
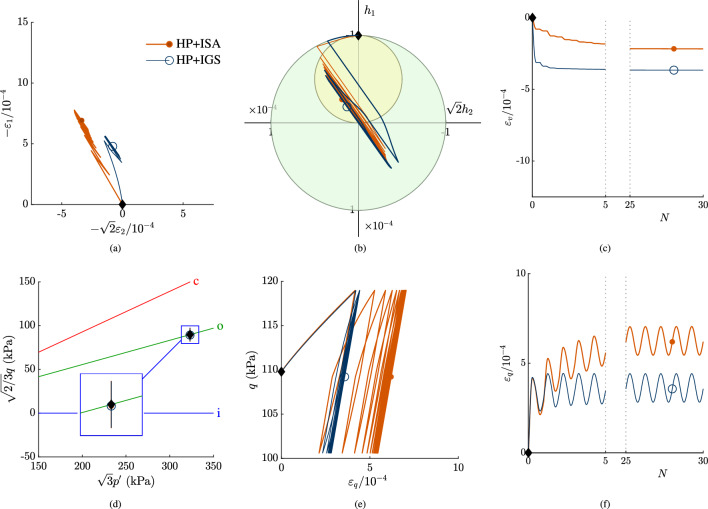
Cyclic uniaxial triaxial tests with $p'=$const, in **a** the Rendulic plane of strain space is shown, in **b** the Rendulic plane of intergranular strain space is shown, where the ISA yield surface corresponds to the initial state. In **c** the volumetric response with ongoing number of cycles is shown. **d** shows the stress path, where **c** indicates the CSL, o the $K_0$-path and i is the hydrostatic axis. **e** is the *q*-$\varepsilon _q$ plot, In **f** the deviatoric strain with ongoing number of cycles *N* is shown

Figure 5 presents a unidirectional ($p'=$ const) test with the same stress increment $\Delta q$ and initial conditions as in Fig. 4. The obtained strain accumulation is lower than for the multiaxial stress loop. This is in accordance with numerical investigations by Poblete et al. [28]. In Fig. 4, the obtained intergranular strain path is a transition between elasticity and hypoplasticity. In the case of $180^\circ$ strain path reversal, there is less accumulation than for the applied stress loop, where the direction of stretching continuously slightly changes. The model’s response is qualitatively confirmed by Ishihara and Yamazaki [16], where it was shown that the obtained strain accumulation for multiaxial tests is higher than for uniaxial loading in simple shear investigations with sand. In addition, in the numerical simulation of the stress loop, artificial accumulation occurs because the elastic model is hypo- and not hyperelastic. A remarkable difference between the models is that HP+IGS presents a higher accumulation of volumetric strain, while it shows less accumulation of deviatoric strain than HP+ISA.

#### Influence of initialization

**Fig. 6:**
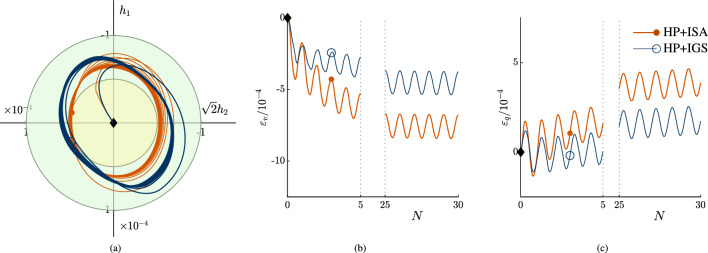
**a** Rendulic plane of intergranular strain space with initial intergranular strain $\varvec{h}_\text {ini}=\textbf{0}$. The other initial conditions coincide with the ones in Fig. 4, $\chi _0=1$ and $\chi _\text {max}=20$ In **b** the volumetric strain with ongoing number of cycles is shown. In **c** the deviatoric strain with ongoing number of cycles *N* is shown

The initialization of the intergranular strain can have a large effect on the final stress or strain accumulation. In the case of Fig. 6, the initial intergranular strain $\varvec{h}_0$ is set to ${0}$. All other initial conditions and applied stress loops coincide with the ones in Fig. 4. The initialization of $\varvec{h}$ affects the response at the very beginning and thus the final accumulation is lower in the case of $\varvec{h}_0=\varvec{0}$ as compared with the fully mobilized intergranular strain in Fig. 4. The initialization of intergranular strain should be considered and carried out thoroughly, as it can have a great impact on the overall result. Furthermore, it seems to have a greater impact on HP+IGS than HP+ISA. This can be observed especially in the accumulation of volumetric strain depicted in Figs. 6b and 4c.

#### Investigations in the deviatoric plane

**Fig. 7:**
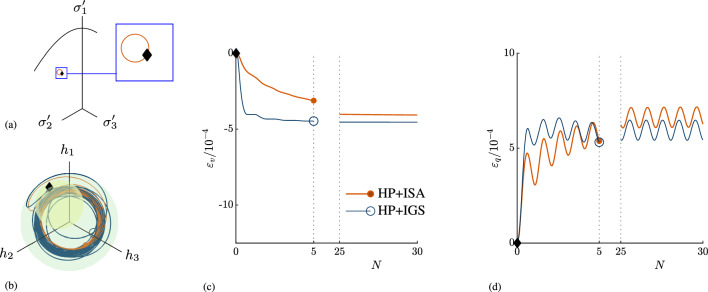
Stress loop in the deviatoric plane **a** is applied in clockwise direction and results in an intergranular strain path, that causes interpolation between elasticity and hypoplasticity in the intergranular strain space in **b**. Therefore, due to the transition from $\chi _0$ to $\chi _\text {max}$, an increase in the number of loading cycles causes a decrease in volumetric and deviatoric strain accumulation, **c** and **d**. The initial conditions are in Table 2. The initial state is marked with $\blacklozenge$ in all plots, in **b** the ISA yield surface corresponds to the initial state

Another case of non-axisymmetric loading conditions presents the application of stress loops in the deviatoric plane with $p'=$const as featured in Fig. 7a. Thereby, the following stress increments were applied in principal stress space:

9 $$\begin{aligned} \Delta \sigma _1'&= \Delta \eta \frac{1}{\sqrt{6}}-\Delta \xi \frac{1}{\sqrt{2}} \end{aligned}$$

10 $$\begin{aligned} \Delta \sigma _2'&= \Delta \eta \frac{1}{\sqrt{6}}+\Delta \xi \frac{1}{\sqrt{2}} \end{aligned}$$

11 $$\begin{aligned} \Delta \sigma _3'&= -\Delta \eta \frac{2}{\sqrt{6}} \end{aligned}$$

whereby $\Delta \xi$ and $\Delta \eta$ are:

12 $$\begin{aligned} \Delta \xi&= r \sin {t} \end{aligned}$$

13 $$\begin{aligned} \Delta \eta&= r \cos {t}. \end{aligned}$$

In the simulation in Fig. 7, $r=5$ kPa and $0\le t\le 2\pi$ are chosen. The initial conditions are summarized in Table 2. The initial stress state $\varvec{T}_0$ is an arbitrary non-axisymmetric stress state with $p'=560$ kPa. The initial intergranular strain that corresponds to this stress state follows from a consolidation path from isotropic state to $\varvec{T}_0$. The applied stress loops in clockwise direction result in a strain path, that causes interpolation between elasticity and hypoplasticity. Therefore, due to the transition from $\chi _0$ to $\chi _\text {max}$, an increase in the number of loading cycles causes a decrease in volumetric and deviatoric strain accumulation rates, see Fig. 7c and d. It is interesting to note that the accumulation with HP+IGS is hardly affected by using lower values of $\chi$, whereas for ISA a reduction in $\chi$ results in an increase in strain accumulation as described above.

### Overshooting

Simulations of cyclic loading can cause unrealistic over- or undershooting effects [2, 5, 25, 42]. Duque et al. [5] showed that with HP+IGS overshooting can be predicted, whereas HP+ISA shows undershooting effects for certain cyclic strain paths. By further investigations and parameter variations, in the following, it is shown that simulations with both models may result in pronounced over- and undershooting. In general, overshooting in the *q*-$\varepsilon _q$ plane occurs if a deviatoric, reloading strain increment causes a higher $\Delta q$, than the deviatoric, unloading strain increment. In the same sense, excessive accumulation of stress (undershooting) occurs if a deviatoric, reloading strain increment causes a lower $\Delta q$, than the one exhibited due to the deviatoric, unloading strain increment.

**Fig. 8:**
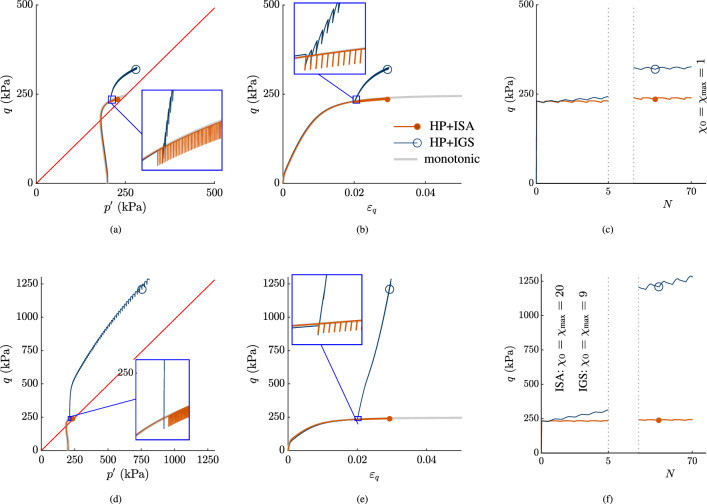
Seventy very small-strain unloading cycles are applied: $0<\Delta \varepsilon _q^\text {unloading}<\sqrt{2/3}R$ with $\Delta \varepsilon _q^\text {unloading}=3\cdot 10^{-5}$ and $\Delta \varepsilon _q^\text {reloading}=1.7\cdot 10^{-4}$: The higher the $\chi _0=\chi _\text {max}$ is, the more pronounced is overshooting for IGS. **a**, **d** stress paths in $p'$-*q* planes, **b**, **e** *q*-$\varepsilon _q$ planes, **c**, **f** *q*-*N* planes

Figures 8 and 10 present simulations of monotonic and cyclic undrained compression tests, whereby both the stress as well as the intergranular strain were initialized as isotropic state (see Table 2).

**Fig. 9:**
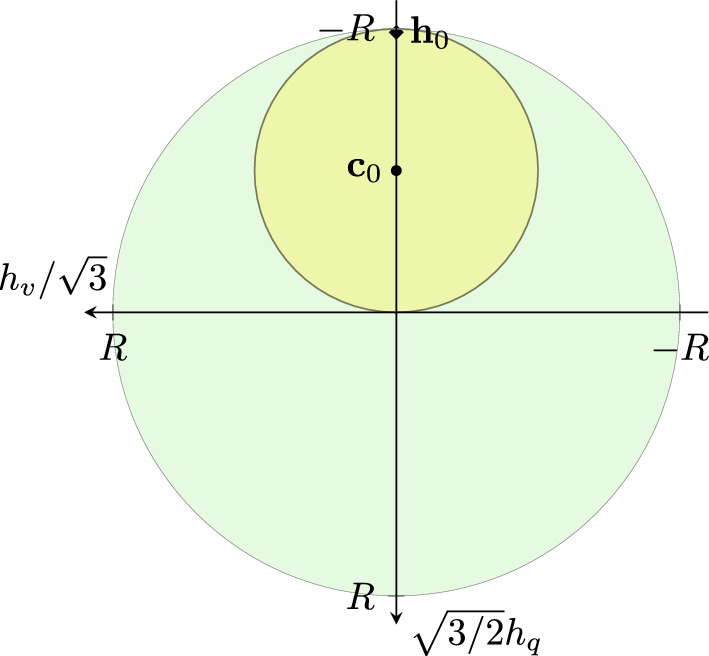
Initial state before cyclic un- and reloading is a fully mobilized intergranular strain state with $h_v=0$ and $h_q=\sqrt{2/3}R$. For HP+ISA, $180^\circ$ strain path reversals then result in purely elastic un- and reloading responses for $\Delta \varepsilon _q<\sqrt{2/3}R$. If, however, the bounding surface is again reached for a larger reloading increment, the behaviour is then purely hypoplastic

**Fig. 10:**
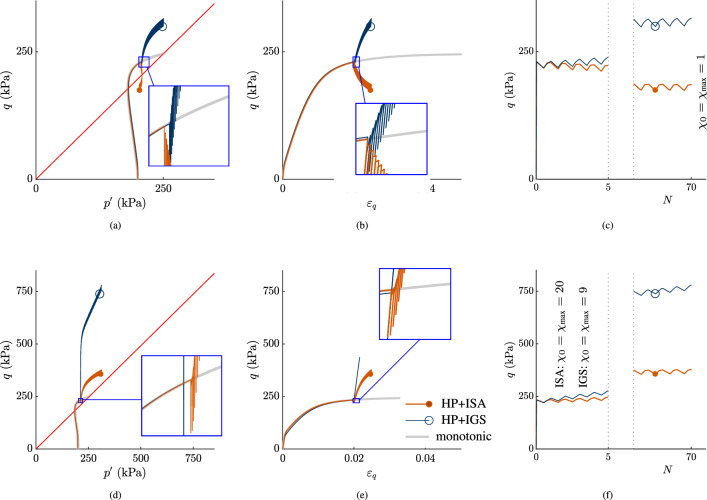
Seventy small-strain unloading cycles are applied with an increased unloading $\Delta \varepsilon _q^\text {unloading}$ compared to Fig. 8: $\sqrt{2/3}R<\Delta \varepsilon _q^\text {unloading}<2\sqrt{2/3}R$ with $\Delta \varepsilon _q^\text {unloading}=1\cdot 10^{-4}$ and $\Delta \varepsilon _q^\text {reloading}=1.7\cdot 10^{-4}$. For both HP+ISA and HP+IGS, the models’ predictions results from transition between elasticity and hypoplasticity. The higher the $\chi _0=\chi _\text {max}$ is, the more pronounced is overshooting. **a**, **d** stress paths in $p'$-*q* planes, **b**, **e** *q*-$\varepsilon _q$ planes, **c**, **f** *q*-*N* planes

The thick, grey line in Figs. 8 and 10 indicates the monotonic loading curve. The numerical samples in the cyclic tests are initially sheared until $\varepsilon _q=0.02$, and then 70 undrained cycles are applied. At cyclic un- and reloading, the initial intergranular strain state reached the fully mobilized isochoric compression state due to the previous monotonic, undrained compression, see Fig. 9.

In Fig. 8, the unloading strain increments $\Delta \varepsilon _q^\text {unloading}=3\cdot 10^{-5}<\sqrt{2/3}R$ with reloading increments $\Delta \varepsilon _q^\text {reloading}=1.7 \cdot 10^{-4}>\Delta \varepsilon _q^\text {unloading}$. In Fig. 8a–c, $\chi _0=\chi _\text {max}$ is set to 1 for both models, while in Fig. 8d–f $\chi _0=\chi _\text {max}=20$ for HP+ISA and $\chi _0=\chi _\text {max}=9$ for HP+IGS.^4^

For HP+IGS, pronounced overshooting effects occur, as the larger reloading strain increment causes a higher $\Delta q$, than the smaller, elastic unloading strain increment. The higher the $\chi _0=\chi _\text {max}$ is, the more pronounced is overshooting. Overshooting effects are reduced by an increased value of $\beta$ and decreased $\gamma _\chi$. However, stress overshooting persists even then. For certain parameters, also undershooting can occur. Then, due to a decrease in stiffness, the larger reloading strain increment causes a lower $\Delta q$, than the smaller, elastic unloading strain increment.

For HP+ISA, the isochoric, small-strain unloading ($\Delta \varepsilon _q^\text {unloading}<\sqrt{2/3}R$) results in an intergranular strain remaining within the purely elastic intergranular strain yield surface (yellow circle in Fig. 9). This implies that the reloading response is purely elastic within this unloading range and then again reaches the boundary surface. Subsequently, after purely elastic reloading within the yield surface, intergranular strain is fully mobilized, and the behaviour is purely hypoplastic. Thus, no over- or undershooting effects are obtained with HP+ISA for undrained strain cycles, as long as $0<\Delta \varepsilon _q^\text {unloading}<\sqrt{2/3}R$.

In Fig. 10, unloading increments $\sqrt{2/3}R<\Delta \varepsilon _q^\text {unloading}=1\cdot 10^{-4}<2\sqrt{2/3}R$ followed by larger reloading increments $\Delta \varepsilon _q^\text {reloading}=1.7\cdot 10^{-4}$ have been applied. *Both* models show pronounced over- or undershooting effects. In Fig. 10a–c, $\chi _0=\chi _\text {max}=1$ for both models, while in Fig. 10d–f, $\chi _0=\chi _\text {max}=20$ for ISA and $\chi _0=\chi _\text {max}=9$ for IGS. As the unloading cycles are within $\sqrt{2/3}R<\Delta \varepsilon _q^\text {unloading}=1\cdot 10^{-4}<2\sqrt{2/3}R$, the unloading and reloading stiffnesses initially follow from elasticity for both models, and are then governed by interpolation between elasticity and hypoplasticity. For the case that the larger reloading strain increment causes a higher $\Delta q$ than the unloading strain increment, overshooting is obtained. For certain parameters, the larger reloading strain increment causes a lower $\Delta q$ than the unloading strain increment, resulting in a decrease in stiffness, undershooting is experienced by ISA. In general, additional investigations have shown that this occurrence becomes more pronounced for lower values of $\gamma _\chi$ (for IGS) and $\chi$, and for higher values of $\beta$. Hence, it can be rendered by both models, even though in Fig. 10 it is depicted only for ISA.

Based on the herein presented analysis, it can be concluded that a boundary in (intergranular) strain space is not sufficient to ban over- or undershooting effects, neither for HP+IGS, nor for HP+ISA.

## Comparison with experimental data

Finally, both models, HP+ISA as well as HP+IGS, are calibrated using the experiments of the highly plastic Lower Rhine Clay (LRC) by Tafili et al. [37]. The index properties of LRC are listed in Table 3. The time-dependent material behaviour of LRC was neglected in the present work, since the studied models do not include it. The calibrated parameters for both models are listed in Table 4. They are determined based on an oedometric normal compression test and several unloading-reloading cycles, monotonic as well as cyclic triaxial tests with variation of initial and loading conditions as will be explained in the following.

**Table 3:** Index properties of Lower Rhine Clay

Material	${w_{L}}\, (\%)$	${w_{P}}\,(\%)$	${I_{P}}\, (\%)$	${\rho _{s}}\, (\text{ g/cm}^3)$	$k\, (\text{ m/s})$
Lower Rhine clay	56.1	22.0	34.0	2.59	$3.6\cdot 10^{-11}$

**Table 4:** Parameters of hypoplasticity with IGS or ISA for LRC

Model	$\varphi _c$	*N*	$\lambda$*	$\kappa$*	$\nu$	$m_T$	$m_R$	*R*	$\beta _R$	$\chi _0$	$\chi _{max}$	$C_a$	$\gamma _\chi$
ISA	$24.2^\circ$	1.298	0.11	0.028	0.2	−	6	$1.2\times 10^{-4}$	0.1	4	20	0.05	−
IGS						4.2	8				9		5.5

### Calibration

**Fig. 11:**
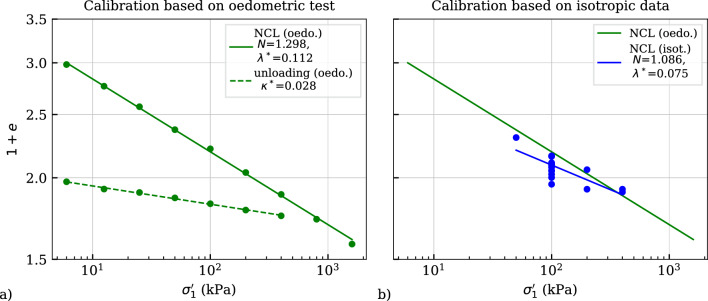
Calibration of *N*, $\lambda$* and $\kappa$* by curve fitting using two methods: **a** oedometric compression test, **b** isotropic consolidation tests

**Fig. 12:**
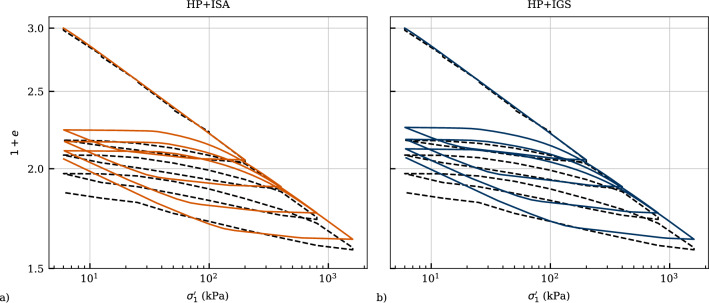
Simulation of an oedometric compression test performed on Kaolin. Parameters calibrated according to Table 4

Figure 11 presents the calibration of the ordinate intercept of the NCL *N* at $p_{ref}'=1$ kPa, the compression index $\lambda ^*$ and the swelling index $\kappa ^*$ considering two methods. Figure 11a comprises an oedometric loading along the normally consolidated line (NCL) and an oedometric unloading path in the $(1+e)$ versus $\sigma _1'$ space. In the same space depicts Fig. 11b the void ratios at the end of primary consolidation at various vertical effective stresses $\sigma _1'$. Both methods can be used for the calibration of the aforementioned parameters as depicted in other works as well Fuentes et al. [9]; Medicus et al. [23]. Apparently, the data of isotropic consolidation tests shows a higher degree of scatter than the oedometric tests, and thus, the parameters denoted with NCL (isot.) obtained by curve fitting vary from those of NCL (oedo.). In order to simulate the oedometric test in Fig. 12, accurately, the parameters obtained by the method depicted in Fig. 11a are used for the simulations in the following.

Figure 12 presents the oedometric test with four unloading-reloading cycles. Both models present similar simulations as the parameters and constitutive equations governing this behaviour are similar and correspond to the fully mobilized state. Hence, the intergranular strain has been initialized fully mobilized in vertical direction for these simulations. Some slight differences are obtained in the hysteretic behaviour of the models, because the parameter $m_R$ influencing the stiffness in unloading is different. Parameters of the intergranular strain models were calibrated based on the undrained cyclic triaxial test LRC-8 with the deviatoric stress amplitude of $q_{ampl}=40$ kPa presented in Sect. 4.3.

**Fig. 13:**
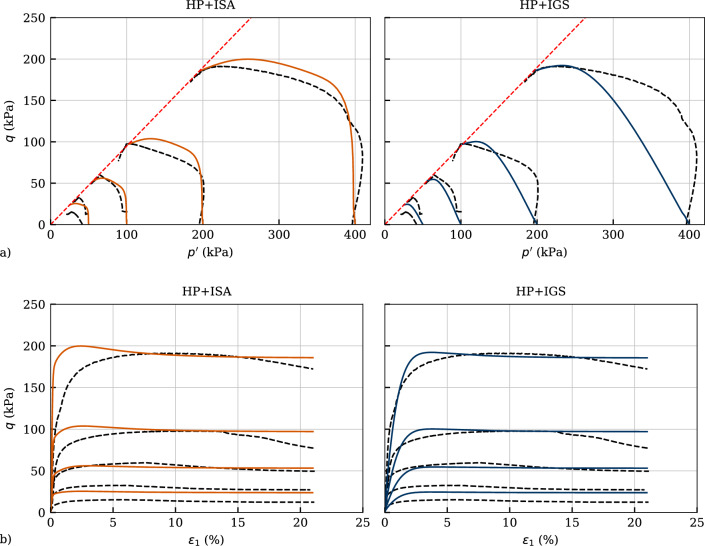
Simulation of monotonic triaxial tests performed on Kaolin with variation of initial mean pressure $p_0'=\{50,\ 100,\ 200,\ 400\}$ kPa. Parameters according to Table 4

Five tests with monotonic triaxial loading are depicted in Fig. 13a in the effective stress space and in Fig. 13b in the deviatoric stress-axial strain space. Two of them (the tests with $p_0'=100$ and 200 kPa) are used for the calibration of the critical friction angle $\varphi _c$ and the Poisson’s ratio $\nu$. The critical friction angle has been calculated using the slope of the triaxial critical state line $M_c=6\sin \varphi _c/(3-\sin \varphi _c)$, which has been drawn connecting the stress ratios with vanishing rate of excess pore water pressure build-up. The Poisson’s ratio has been fitted to match the initial stiffness in the $q-\varepsilon _1$ space. The remaining two experiments with $p_0'=\{50,\ 400\}$ kPa (whereby two experiments are conducted with the same initial mean effective pressure of $p_0' \approx 50$ kPa resulting in only one simulation) have been simulated to validate the calibrated parameters. The intergranular strain has been initialized in fully mobilized isotropic direction, i.e. $h_{ii}=-R/\sqrt{3}$. Even though the models formulations under fully mobilized intergranular strain obey equal constitutive relations, a significant difference can be observed between the simulations. Hereby, a $90^\circ$ strain path reversal occurs, hence undrained triaxial compression is performed after isotropic compression. As can be concluded from the qualitative cu test in Fig. 1 as well as pointed out in detail in [34], the shear modulus degradation of IGS under these conditions takes place faster than the one of ISA. A reason for this is that IGS postulates a slightly increased stiffness with $m_T<m_R$ for ‘neutral’ strain rate ($\varvec{D}\perp \varvec{h}^0$ or $\varvec{D}:\varvec{h}^0=O$). On the other hand, in ISA, only $m_R$ is used, see Table 4. Therefore, the initial stiffness is overestimated as shown in Fig. 13b and is overestimated by HP+ISA, while HP+IGS succeeds on its reproduction. Otherwise, the effective stress paths are more accurately described by HP+ISA.

### Validation with monotonic triaxial tests on overconsolidated samples

**Fig. 14:**
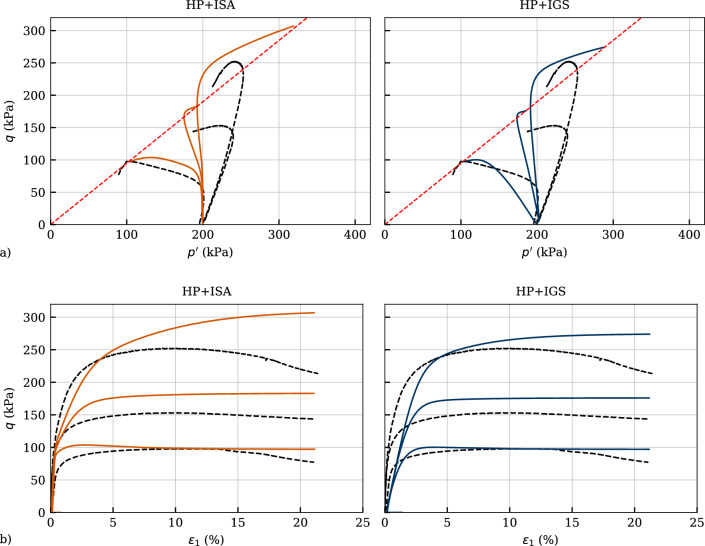
Simulation of monotonic triaxial tests performed on Kaolin with variation of initial overconsolidation ratio OCR$_0=\{1,\ 2,\ 4\}$. Parameters according to Table 4

The models have been validated in the following sections through simulations of undrained triaxial tests with different initial overconsolidation ratios as well as with cyclic triaxial tests with variation of deviatoric stress amplitude. Figure 14 presents a comparison between simulations and experiments under undrained shearing with OCR$_0=\{1,\ 2,\ 4\}$. The test with OCR$_0=1$ is depicted in Fig. 4 and for the sake of completeness is shown here as well. Even though HP+IGS reproduces the shear strength for OCR$_0=4$ more accurately, both models substantially overestimate the overall shear strength for initially overconsolidated samples. This may be attributed to the fact, that initially overconsolidated laboratory sample require higher strains to reach the critical state. At these strains, however, a homogeneous deformation distribution inside the specimen cannot be guaranteed. On the other hand, HP+ISA renders more accurate description of the initial stiffness (see Fig. 14b) and may thus perform better in a settlement analysis accounting for overconsolidated clays. The underestimation of initial tangent stiffness with HP+IGS, as already observed in the first qualitative simulations in Fig. 1 and in the calibration depicted in Fig. 13 is present for OCR$_0>1$ in Fig. 14 as well.

### Validation with cyclic triaxial tests

Five cyclic triaxial tests with variation of the deviatoric stress amplitude between $q_{ampl}=\{60,\ 45,\ 40,\ 35,\ 25\}$ kPa have been simulated and are shown in the following. As pointed out in Tafili et al. [37], the loading direction was changed once the specified stress amplitude was reached whether in compression or in extension. The initial stress of all simulations was $\sigma _{ii}=-5$ kPa, and the initial intergranular strain tensor amounted $\varvec{h}=\varvec{0}$. Hence, the isotropic preloading was modelled by both models, and afterwards the cyclic loading has been applied.

**Fig. 15:**
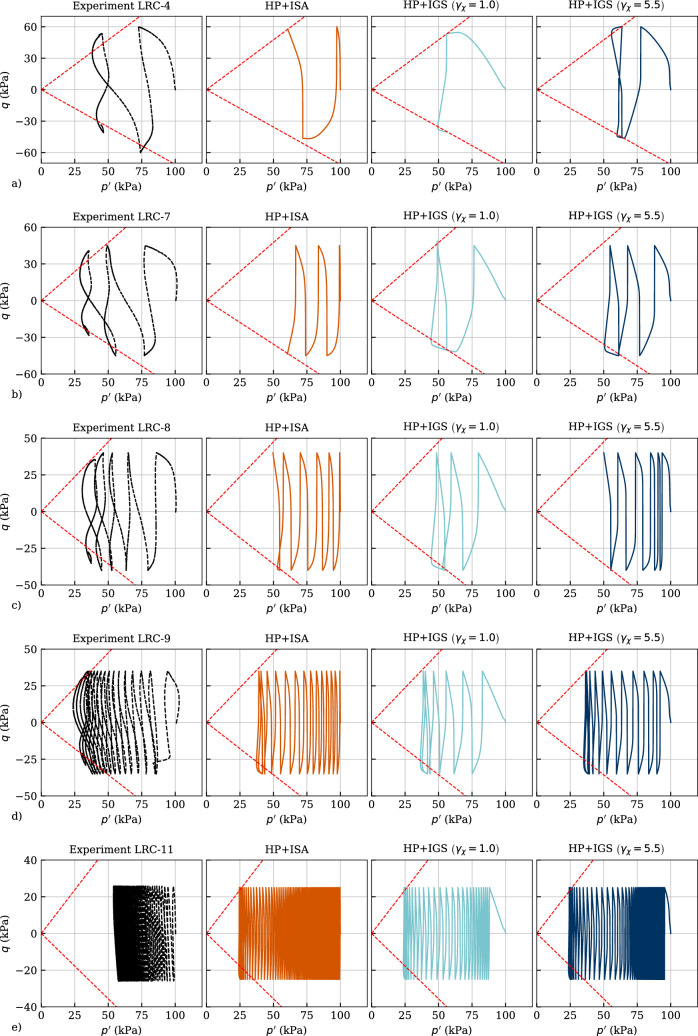
Simulation of cyclic triaxial tests performed on Kaolin with variation of deviatoric stress amplitude $q_{ampl}=\{60,\ 45,\ 40,\ 35,\ 25\}$ kPa in *q* versus *p* space. Parameters according to Table 4, whereas $\gamma _chi=1$ converts IGS to the original version of Niemunis and Herle [27]

**Fig. 16:**
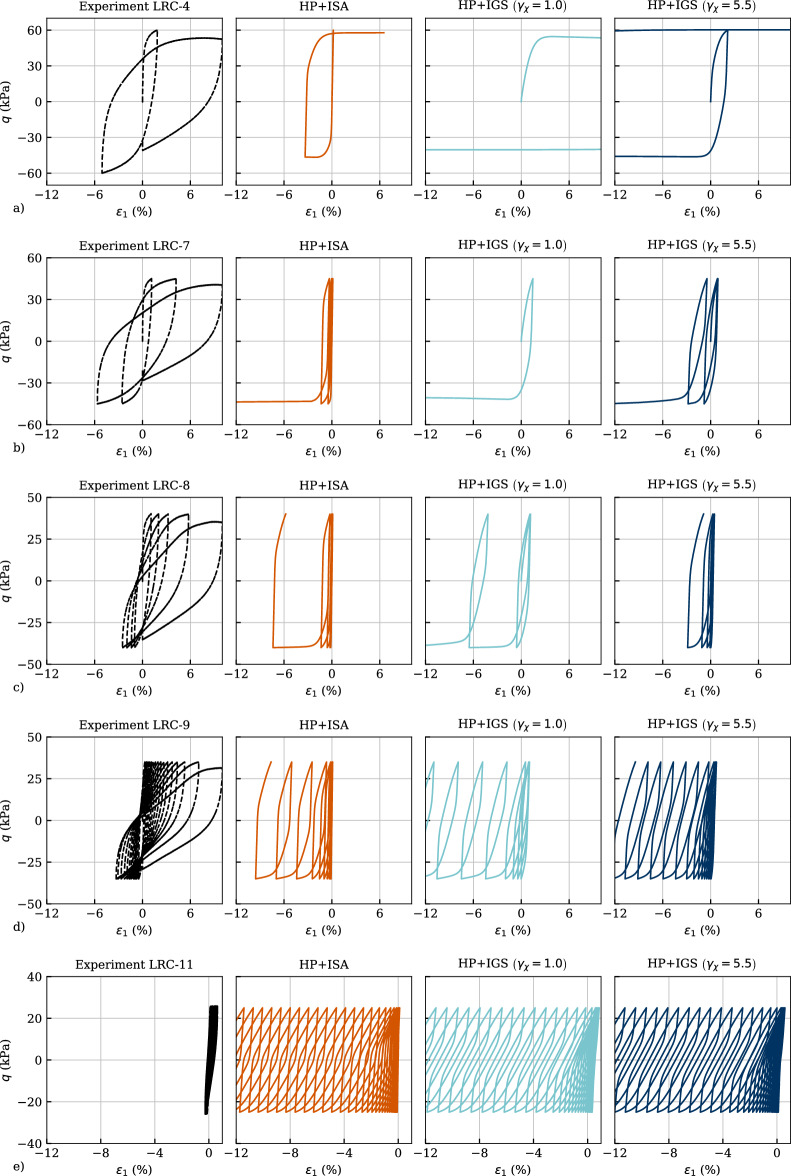
Simulation of cyclic triaxial tests performed on Kaolin with variation of deviatoric stress amplitude $q_{ampl}=\{60,\ 45,\ 40,\ 35,\ 25\}$ kPa in *q* versus $\varepsilon _1$ space. Parameters according to Table 4, whereas $\gamma _chi=1$ converts IGS to the original version of Niemunis and Herle [27]

**Fig. 17:**
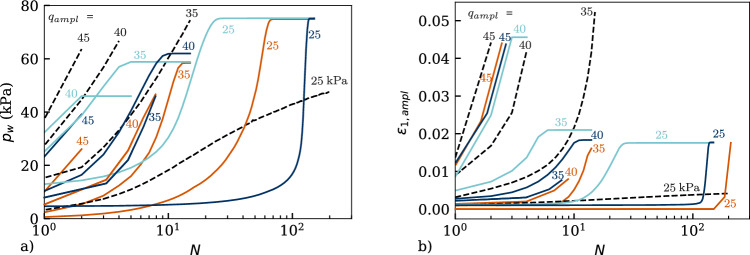
Simulation of cyclic triaxial tests performed on Kaolin with variation of deviatoric stress amplitude $q_{ampl}=\{60,\ 45,\ 40,\ 35,\ 25\}$ kPa in **a** excess pore water pressure $p_w$ versus number of cycles *N*, **b** axial strain amplitude $\varepsilon _{1,ampl}$ versus number of cycles *N*. Parameters according to Table 4

Figure 15 depicts all comparisons in the effective stress space, whereas Fig. 16 presents them in the $q-\varepsilon _1$ space. In order to see the influence of the parameter $\gamma _\chi$, it is varied between $\gamma _\chi =1.0$ and $\gamma _\chi =5.5$ in the simulations with HP+IGS. Figure 15 indicates a good reproduction of the mean effective stress reduction with the number of cycles until the cyclic mobility, which in the experiment is reached with an eight-shaped effective stress path. This cannot be reproduced by either the simulation with HP+ISA nor with HP+IGS. Furthermore, a closer look in the excess pore water pressure $p_w$ versus the number of cycles *N* curves depicted in Fig. 17a reveals that the trend is not accurately reproduced by none of the models. While the HP+IGS without extension (II) ($\gamma _\chi =1.0$) overestimates the accumulation rates of the excess pore water pressure for any test, to some extent, more realistic results are provided by HP+IGS (considering extension (I) and (II)) and HP+ISA. It should be noted that five different amplitudes are simulated with the same set of parameters. Thereby, especially HP+ISA shows a reduction in the rate of increase in $p_w$ with decreasing amplitude. However, all models simulate a too steep $p_w$ versus *N* curve.

In Fig. 16, the deviatoric stress versus axial strain relations are presented for the experiments and the simulations. For $q_{ampl}=60$ kPa (Fig. 16a) at the first quarter of the second cycle, where reloading is applied in triaxial compression, the prescribed amplitude cannot be reached even by increasing the axial strain, hence deformation flow of the sample takes place. While HP+IGS with $\gamma _\chi =1.0$ shows deformation flow at the very first quarter of the first cycle, HP+ISA and HP+IGS with $\gamma _\chi =5.5$ reproduce well the behaviour observed in the experiment. Even though the direction of accumulation observed in the experiments in triaxial compression is not well described by the models, the magnitude of accumulation of axial strain is well captured by HP+ISA and to a certain extent also by HP+IGS with $\gamma _\chi =5.5$ for $q_{ampl}\ge 35$ kPa. For the lowest deviatoric amplitude of $q_{ampl}=25$, kPa all models significantly overestimate the axial strain accumulation. It should be noted that more than 100 cycles have been simulated herewith. Furthermore, the experimental results showed an increasing double strain amplitude during cyclic mobility. The models were not able to reproduce this behaviour, and instead, reproduced a bias in the strain accumulation towards extension. This restricts the models’ application to boundary value issues where the behaviour at large deformations is significant, e.g. earthquake loading or tailings dam.

In Fig. 17b, the amplitudes of axial strain versus the number of cycles are evaluated. As expected from Fig. 16a for the deviatoric amplitude of $q_{ampl}=60$ kPa, no cycle could be applied, neither in the experiments nor in the simulations. For HP+IGS with $\gamma _\chi =1.0$, the same holds for $q_{ampl}=45$ kPa, while both HP+ISA and HP+IGS with $\gamma _\chi =5.5$ underestimate the axial strain amplitude for the same number of cycles for all variations of deviatoric stress. In contrary, HP+IGS with $\gamma _\chi =1.0$ overestimates the rate of the axial strain amplitude with the number of cycles, which is expected due to the too fast pore pressure build-up represented in Fig. 17a.

## Summary and conclusion

In this article, clay hypoplasticity is coupled with both small-strain extensions ISA and intergranular strain. Detailed investigations are shown and compared for both models comprising qualitative analysis addressing strengths and limitations of the respective equations and comparisons of the models’ predictions with experimental data. The comparisons contain oedometric, monotonic as well as cyclic triaxial tests with varying loading and initial conditions. The findings can be summarized as follows:

(i) The strain accumulation may be decreased with an increasing number of cycles with HP+IGS due to the development from $\chi _0$ to $\chi _\text {max}$.
(ii) Both models experience pronounced stress over- and undershooting for certain cyclic loading paths and parameter values. A boundary in (intergranular) *strain* space is thus not sufficient to ban these effects.
(iii) The model parameters has been calibrated based on an oedometric test, monotonic triaxial tests conducted on initially normally consolidated samples as well as two cyclic triaxial tests of Lower Rhine clay. The remaining tests with overconsolidated initial states of the samples as well as variation of deviatoric cyclic stress amplitude has been used for validation purposes. Comparisons with the laboratory data under monotonic loading have shown that the relaxation of the mean effective stress is initially overestimated with HP+IGS, even though both models obey hypoplasticity for clay under fully mobilized states. Simulations of cyclic triaxial tests have shown that both models are able to reproduce qualitatively the decrease in excess pore water pressure accumulation rate with increasing number of cycles and the influences of the deviatoric stress loading amplitude. However, even though improved compared to the original IGS formulation, the predicted accumulations of strains show a bias towards extension.

## Data Availability

The data will be made available upon request.

## Appendix A: Symbols and notations

In this article, the symbolic notation is used for the effective Cauchy stress $\varvec{T}$ and stretching $\varvec{D}$, but in some cases the more familiar symbol $\sigma _i'$ instead of $T_i$ is used for the principal stresses. For the principal components of stress, compression is defined negative. Tensors are written in bold capital letters (e.g. $\varvec{X}$). $||\varvec{X}||=\sqrt{{{\,\textrm{tr}\,}}\varvec{X}^2}$ is the Frobenius norm of $\varvec{X}$, ${{\,\textrm{tr}\,}}\varvec{X}$ is the sum of the diagonal components of $\varvec{X}$. The superscript 0 indicates a normalized tensor, i.e. $\varvec{X}^0 = \varvec{X}/||\varvec{X}||$. Stresses are considered as effective ones. $\mathring{\varvec{T}}$ is the co-rotational, objective stress rate. The stretching tensor $\varvec{D}$ is the symmetric part of the velocity gradient.

The void ratio *e* is the ratio of the volume of the voids $V_p$ to the volume of the solids $V_s$. Note that with $p'=-\frac{1}{3}{{\,\textrm{tr}\,}}\varvec{T}$, the mean effective stress is positive for compression. $\varepsilon _{\text {v}}={{\,\textrm{tr}\,}}\varvec{\varepsilon }$ is the volumetric strain. For compressive strain, $\varepsilon _i$ is defined negative.

The deviatoric stress *q* for rectilinear extensions is

$$\begin{aligned} \begin{aligned} q&= \sqrt{\frac{1}{2}[(\sigma _{22}'-\sigma _{33}')^2+(\sigma _{33}'-\sigma _{11}')^2+(\sigma _{11}'-\sigma _{22}')^2]} \end{aligned} \end{aligned},$$

and the deviatoric strain reads $\varepsilon _q=\sqrt{2/9\left( (\varepsilon _1-\varepsilon _3)^2+(\varepsilon _3-\varepsilon _1)^2+(\varepsilon _1-\varepsilon _2)^2 \right) }$. For axisymmetric conditions the Rendulic plane is often used. For a conventional triaxial compression or oedometric compression test, the axial stress is denoted with $\sigma _1'$ and the radial stress is denoted with $\sigma _2' (=\sigma _3')$. The associated strains are $\varepsilon _1$ and $\varepsilon _2=\varepsilon _3$.

Initial values are labelled with the subscript 0. Any symmetric second-order tensor can be written as vector with the principal values $\varvec{X}_\text {v} = \left[ X_1,X_2,X_3\right]^\text{T}$. We use this to display tensors in figures, however, we do not use the notation $\varvec{X}_\text {v}$, as it is implicitly clear that $\varvec{X}$ is shown as a vector in these figures. Bold calligraphic letters denote tensors of 4^th^ order (e.g. $\pmb {\mathcal {M}}$). We use different kinds of tensor operations employing the Einstein summation convention. In particular, the indices follow the lexicographic order: $(\varvec{X}\otimes \varvec{Y})_{ijkl}= X_{ij}Y_{kl}$, $\varvec{X}\mathbin {:}\varvec{Y}= X_{ij}Y_{ij}$ and $(\pmb {\mathcal {L}}\mathbin {:}\varvec{D})_{ij}= \mathcal {L}_{ijkl}D_{kl}$. We employ the unit tensor of second-order $\varvec{I}$ with $I_{ij} = \delta _{ij}$.

## Footnotes

1 The objective stress rate is then given by $\mathring{\varvec{T}}=\pmb {\mathcal {M}}:\varvec{D}$.

2 Note that $y_h$ is often denoted as $\rho$ within the intergranular strain framework. In order to keep the notation between ISA and IGS as consistent as possible, we stick to $y_h$ in this work.

3 If the principal components of the tensors $\varvec{h}$ and $\varvec{D}$ are interpreted as vectors, and they point in the opposite direction, then $\varvec{h}:\varvec{D}<0$. If they point in the same direction, then $\varvec{h}:\varvec{D}>0$.

4 In Fig. 2, it was shown that for these values of $\chi _0/\chi _\text {max}$, the best concordance between the models’ simulations was achieved.
